# Implementation Outcome Scales for Digital Mental Health (iOSDMH): Scale Development and Cross-sectional Study

**DOI:** 10.2196/24332

**Published:** 2021-11-23

**Authors:** Natsu Sasaki, Erika Obikane, Rajesh Vedanthan, Kotaro Imamura, Pim Cuijpers, Taichi Shimazu, Masamitsu Kamada, Norito Kawakami, Daisuke Nishi

**Affiliations:** 1 Department of Mental Health Graduate School of Medicine The University of Tokyo Tokyo Japan; 2 Department of Population Health Grossman School of Medicine New York University New York, NY United States; 3 Department of Clinical, Neuro and Developmental Psychology Vrije Universiteit Amsterdam Amsterdam Netherlands; 4 Behavioral Science Division Center for Public Health Sciences National Cancer Center Tokyo Japan; 5 Department of Health and Social Behavior Graduate School of Medicine The University of Tokyo Tokyo Japan

**Keywords:** implementation outcomes, acceptability, appropriateness, feasibility, harm

## Abstract

**Background:**

Digital mental health interventions are being used more than ever for the prevention and treatment of psychological problems. Optimizing the implementation aspects of digital mental health is essential to deliver the program to populations in need, but there is a lack of validated implementation outcome measures for digital mental health interventions.

**Objective:**

The primary aim of this study is to develop implementation outcome scales of digital mental health for different levels of stakeholders involved in the implementation process: users, providers, and managers or policy makers. The secondary aim is to validate the developed scale for users.

**Methods:**

We developed English and Japanese versions of the implementation outcome scales for digital mental health (iOSDMH) based on the literature review and panel discussions with experts in implementation research and web-based psychotherapy. The study developed acceptability, appropriateness, feasibility, satisfaction, and harm as the outcome measures for users, providers, and managers or policy makers. We conducted evidence-based interventions via the internet using UTSMeD, a website for mental health information (N=200). Exploratory factor analysis (EFA) was conducted to assess the structural validity of the iOSDMH for users. Satisfaction, which consisted of a single item, was not included in the EFA.

**Results:**

The iOSDMH was developed for users, providers, and managers or policy makers. The iOSDMH contains 19 items for users, 11 items for providers, and 14 items for managers or policy makers. Cronbach α coefficients indicated intermediate internal consistency for acceptability (α=.665) but high consistency for appropriateness (α=.776), feasibility (α=.832), and harm (α=.777) of the iOSDMH for users. EFA revealed 3-factor structures, indicating acceptability and appropriateness as close concepts. Despite the similarity between these 2 concepts, we inferred that acceptability and appropriateness should be used as different factors, following previous studies.

**Conclusions:**

We developed iOSDMH for users, providers, and managers. Psychometric assessment of the scales for users demonstrated acceptable reliability and validity. Evaluating the components of digital mental health implementation is a major step forward in implementation science.

## Introduction

### Background

Due to rapid advances in technology, mental health interventions delivered using digital and telecommunication technologies have become an alternative to face-to-face interventions. Digital mental health interventions vary from teleconsultation with specialists (eg, physicians, nurses, psychotherapists) to fully or partially automated programs led by web-based systems or artificial intelligence [1,2]. For example, internet-based cognitive behavioral therapy has been found useful for improving depression, anxiety disorders, and other psychiatric conditions [3-5]. Moreover, a recent meta-analysis suggested that internet-based interventions were effective in preventing the onset of depression among individuals with subthreshold depression, indicating future implications for community prevention [6]. Past studies have demonstrated that mental health interventions are suitable for digital platforms because of several reasons: rare need for laboratory testing of patients, chronic shortage of human resources in the field of mental health, and stigma often experienced by patients in consulting mental health professionals [7].

Although numerous studies have demonstrated the efficacy of digital mental health interventions, many people do not benefit from them mainly due to insufficient implementation. Implementation is defined as “a specified set of activities designed to put into practice a policy or intervention of known dimensions” [8]. The entire care cascade can benefit from optimization. People with mental health problems are known to face psychological obstacles to treatment [9] due to lack of motivation [9,10], lower mental literacy [11], or stigma [12]. Moreover, digital mental health interventions face high attrition and low adherence to programs especially in open-access websites [13-15]. This may be because implementation aspects have not been fully examined when the interventions are being developed. One of the major barriers is the lack of reliable and valid process measures. Validated measures are needed to monitor and evaluate implementation efforts. Core implementation outcomes include acceptability, appropriateness, feasibility, adoption, penetration, cost, fidelity, and sustainability [16,17]. However, most of these measures have not yet been validated. Weiner et al have developed validated scales for acceptability, appropriateness, and feasibility [18], but these scales were not designed for digital mental health settings. A systematic review of implementation outcomes in mental health settings reported that most outcomes focused on acceptability, and other constructs were underdeveloped without psychometric assessment [19].

Moreover, implementation involves not only the patients targeted by an intervention but also individuals or groups responsible for program management, including health care providers, policy makers, and community-based organizations [8]. Providers have direct contact with users. Managers or policy makers have the authority to decide on the implementation of these programs.

### Objectives

To our best knowledge, outcome measurements to evaluate implementation aspects concerning users, providers, and managers or policy makers are not available in digital mental health research. Therefore, the primary aim of this study is to develop new implementation outcome scales for digital mental health (iOSDMH) interventions that can be applied for users, providers, and managers or policy makers. The secondary aim is to validate the implementation scale for users. This study does not include validation of the implementation scale for providers and managers because the study does not involve providers and managers.

## Methods

### Study Design

We originally developed the English and Japanese versions of the iOSDMH based on previously published literature [18,19], which proposed the 3 measures of the implementation outcome scale and provided a systematic review of implementation outcomes. The development of iOSDMH consisted of 3 phases. In the first phase, literature review on implementation scales was conducted, and scales with high scores for evidence-based criteria were selected for further review. Each item from the item pool was critically reviewed by 3 researchers, and they discussed whether the items were relevant for digital mental health. Based on the selected items, the team developed the first drafts of the scales for users, providers, and managers or policy makers. In the second phase, the draft of the iOSDMH was carefully examined by 2 implementation researchers and 1 mental health researcher. With these expert panels, the research team discussed the relevance of the selected items in each category as well as the wording of each question and created the second drafts of the scales. In the third phase, the draft of the iOSDMH was presented to the implementation and digital mental health researchers to confirm the scales and further changes were made based on their inputs. After confirming the relevance of the scales with the expert panels, we conducted an internet-based survey to examine the scale properties of the Japanese version of the iOSDMH for users. Although the iOSDMH targeted 3 categories of implementation stakeholders, namely users, providers, and managers or policy makers [8], tool validation was conducted for users only, as the study did not involve providers and managers.

### Ethical Considerations

This study was approved by The Research Ethics Committee of the Graduate School of Medicine/Faculty of Medicine, University of Tokyo (No. 2019361NI). The aims and procedures of the study were explained on the web page before participants answered the questionnaire. Responses to the questionnaire were considered as the consent to participate.

### Development Process of iOSDMH

The development of the iOSDMH consisted of 3 phases. In the first phase, 3 of the investigators (EO, NS, and DN) reviewed 89 implementation scales from previous literature and a systematic review of implementation outcomes [18,19]. After the review, we selected 9 implementation scales (171 items) that were rated with evidence-based criteria in the following categories: acceptability of the intervention process, acceptability of the implementation process, adoption, cost, feasibility, penetration, and sustainability. Each item was reviewed carefully by 3 researchers, and 4 highly scored instruments in terms of psychometric and pragmatic quality were selected [20-23]. The following concepts were considered relevant in measuring implementation aspects of digital mental health interventions. Moore et al [21] developed the assessment tool for adoption of technology interventions. Whittingham et al [22] evaluated the acceptability of the parent training program. Hides et al [20] reported the feasibility and acceptability of mental health training for alcohol and drug use. Yetter [23] reported the acceptability of psychotherapeutic intervention in schools. Relevant items were adapted for the web-based mental health interventions, and those not relevant in the context of digital mental health were excluded.

The iOSDMH consisted of two parts: (1) evaluations and (2) adverse events of using digital mental health programs.

In the second phase, the drafts of the iOSDMH for users, providers, and managers were reviewed by experts on web-based psychotherapy (KI) and implementation science (MK and RV), and a consensus was reached to categorize all items into the concepts of acceptability, appropriateness, and feasibility for evaluation. We primarily had 22 items for evaluating the use of digital mental health programs and 6 adverse events of the program for users. We narrowed these to 14 items for evaluations and 5 items for adverse events following discussions with expert panels. For the iOSDMH of providers, we first had 14 items for evaluations and 1 item for adverse events; we then selected 10 items for evaluations and 1 item for adverse events. For the iOSDMH of managers, we first had 11 items for evaluations and 1 item for adverse events but changed them to 13 items for evaluations and 1 item for adverse events. Acceptability is the perception that a given practice is agreeable or palatable, such as feeling “I like it.” Wordings of the items on acceptability (Items 1, 2, and 3 for users, and Item 2 for managers) were taken from Moore et al [21]. Item 3 for users and Items 1, 3, and 4 for managers were from Whittingham et al [22]. The wording of Item 4 for providers was from Yetter et al [23]. Appropriateness is the perceived fit, relevance, or compatibility, such as feeling “I think it is right to do.” Wordings of Item 5 for users, and Items 5 and 7 for managers were from Moore et al [21]. The wording of Item 8 for providers was based on Hides et al [20]. Items 4, 6, and 7 for users and Item 6 for managers were originally developed based on discussions. Item 9 for providers and Item 8 for managers were worded according to Whittingham et al [22]. Feasibility is the extent to which a practice can be successfully implemented [17]. Wordings of Items 8 and 9 for users, Item 7 for providers, and Item 12 for managers were from Moore et al [21]. Items 10, 11, and 13 for users and Item 8 for providers were from Hide et al [20]. Items 12 and 14 for users, Item 9 for providers, and Items 9, 10 and 11 for managers were originally developed based on discussions. In addition to the 3 concepts, we added 1 item related to overall satisfaction in the evaluation section because overall satisfaction is considered important in implementation processes [17]. Previous literature distinguished between satisfaction and acceptability, with acceptability being a more specific concept referring to a certain intervention and satisfaction usually representing general experience [16]. However, we considered that overall satisfaction was an important client outcome of process measures. The second part involved harm (ie, adverse effects of interventions). Burdens and adverse events in using digital programs should be considered because digital mental health interventions are not harm free [24].

In the final step, the second drafts of the iOSDMH for users, providers, and managers were reviewed by 2 external researchers (PC and TS), 1 digital mental health researcher, and 1 implementation researcher, and corrections were made based on discussions. We recognized that the relevance of some items differed according to cultural contexts of responders. For example, Item 2 on acceptability for users, Item 3 on acceptability for providers, and Item 2 on acceptability for managers asked whether using the program would improve their social image, or their evaluation of themselves or their organizations. Improving social image may be important and beneficial in some cultural groups but not as much in others. Researchers of 3 different countries considered these items to be relevant, and therefore, we preserved these items. All coauthors engaged in a series of discussions until a consensus was reached on whether the items reflected the appropriate concepts, as well as the overall comprehensiveness and relevance of the scale. None of the objective criteria was adopted in the process of reaching consensus.

**Figure 1:**
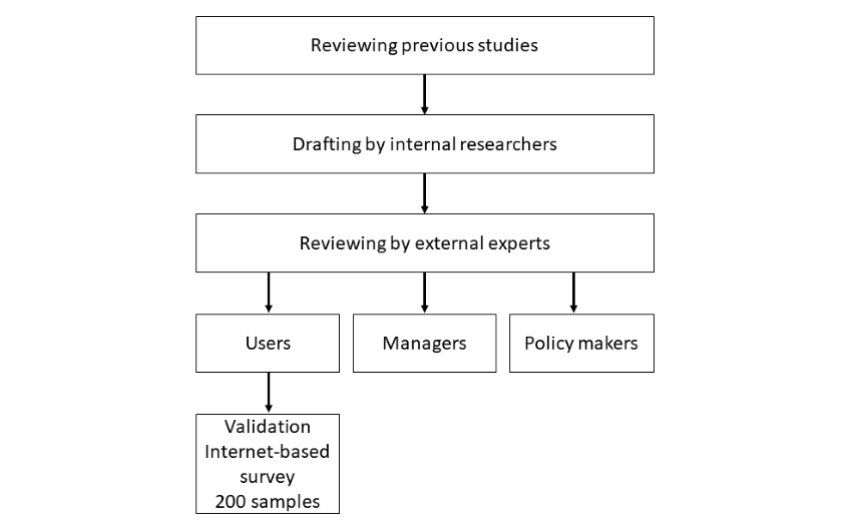
Development process of the implementation outcome scales for digital mental health.

The iOSDMH was developed for targeting 3 different groups that are involved in the implementation process: users (ie, patients), providers, and managers or policy makers. Providers are people who have direct contact with users (eg, medical: nurse; workplace: person in charge). Managers or policy makers are people who have authority to decide on the implementation of this program (eg, responsible person). These scales did not restrict the study settings (eg, clinic workplace, and school). For example, the implementation of workplace-based interventions may involve workers (users), human resource staff (providers), and company owners (policy makers). Moreover, these scales aimed to evaluate the implementation aspects related to users, providers, and managers after the users completed or at least partially received the internet-based intervention. Most items were developed assuming that the users had prior experience in receiving the internet-based intervention. The process of developing the iOSDMH is shown in Figure 1.

### Internet-Based Survey

Participants were recruited on an internet-based crowd working system (CrowdWorks, Inc), which has more than 2 million registered workers. The criterion for eligibility was to be over 20 years old. Participants were required to learn from the self-help information website UTSMeD [25], a digital mental health intervention. The UTSMeD website was developed to help Japanese general workers cope with stress and depression. It contains self-learning psychoeducational information on mental health (eg, stress management). This web-based UTSMeD intervention has proven effective in reducing depressive symptoms and improving work engagement among Japanese workers in previous randomized controlled trials [26,27]. In our study, participants were asked to explore the UTSMed website for as long as they liked and take quizzes on mental health. They answered the Japanese version of the iOSDMH for users (14 items in 2 pages) after they received acceptable scores (ie, 8 or more of 10 questions answered correctly) in the quizzes. The participants received web-based points as incentives for participation. As the current UTSMeD is an open-access website and authors directly provided the URL to participants, the study did not involve any providers, managers, or policy makers. The psychometric assessment thus was limited to users. Gender, age, marital status, education attainment, income, work status, occupation type, and employment contract constituted the demographic information. The target sample size was determined as 10 times the number of items needed to obtain reliable results (eg, 200 participants). The survey was conducted through the internet-based crowd working system. Completed answers were obtained without missing variables.

### Statistical Analysis

To assess the internal consistency of the Japanese iOSDMH, Cronbach α coefficients were calculated for all scales and each of the 4 subscales (acceptability, appropriateness, feasibility, and harm). To assess structural validity, exploratory factor analysis (EFA) was conducted because previous studies have shown that acceptability and appropriateness are conceptually similar [16,18]. EFA was conducted by excluding 1 item of overall satisfaction, as the concept of satisfaction cannot be applied to each of the 4 subscales. We extracted factors with eigenvalues of more than 1, following the Kaiser–Guttman “eigenvalues greater than one” criterion [28], using the least-squares method with Promax rotation. Items with factor loadings above 0.4 were retained [29].

Statistical significance was defined as *P*<.05. All statistical analyses were performed using the Japanese version of SPSS 26.0 (IBM Corp).

## Results

### Development of iOSDMH

The final version of the iOSDMH for users contained 3 items for acceptability based on Moore and Whittingham [21,22] and 4 items for appropriateness, 1 of which was based on Moore [21]. The others were original; there were 6 items for feasibility, 5 of which were based on Moore and Hide [20,21], and 1 item was original; we developed 5 original items for harm and 1 for overall satisfaction. The iOSDMH states “Please read the following statements and select ONE option that most describes your opinion about the program.” The response to each item was scored on a 4-point Likert-type scale ranging from 1 (disagree) to 4 (agree). The iOSDMH for providers and managers or policy makers has an option 5 (don’t know). Details are provided in Multimedia Appendix 1.

The final version of the iOSDMH for providers contained 3 items for acceptability, 2 of which were based on Yetter [23], and 1 item was original; 3 items for appropriateness, 2 of which were based on Yetter [23], and 1 item was original; 3 items for feasibility, 1 of which was original and 2 were based on Moore [21] and Hides [20]; 1 original item for harm; and 1 for overall satisfaction. For acceptability, Item 1 evaluated the providers’ perceived acceptance of the program for protecting the mental health of its users, whereas Items 2 and 3 focused on their own acceptability to implement the program in their workplace. For appropriateness, Items 4 and 6 asked about the providers’ perceived appropriateness of the program for users, whereas Item 5 asked about the appropriateness of the program considering the situation of the providers. For feasibility, Item 7 evaluated the providers’ perception of the program’s feasibility for users, and Items 8 and 9 focused on the willingness of providers to provide the program to users.

The final version of the iOSDMH for managers or policy makers contained 4 items for acceptability, 3 of which were based on Whittingham [22] and the other on Moore [21]; 4 items were for appropriateness, 2 of which were based on Moore [21]; 1 item was based on Whittingham [22], and the other one was original; there were 4 items for feasibility, 1 of which was based on Moore [21] and the others were original; we had 1 original item for harm and 1 item for overall satisfaction. Similar to the iOSDMH for providers, each factor of the scale contained questions on managers’ perceptions on implementation in terms of the conditions of users and providers, as well as the managers themselves. For example, Items 1 and 2 asked about the acceptability of the program for the institution, whereas Item 3 focused on managers’ perceived acceptability for providers, and Item 4 evaluated managers’ perceived acceptability for users. For appropriateness, Items 5 and 7 focused on the appropriateness of the program for the institution, and Item 8 assessed the appropriateness of the program for users according to managers’ perceptions. For feasibility, Items 9 and 10 examined the feasibility of the program for the institution as perceived by managers or policy makers. Item 11 evaluated managers’ perceived feasibility for providers, and Item 12 evaluated managers’ perception of feasibility for users.

### Internet-Based Survey

We recruited 200 participants, whose characteristics are presented in Table 1. Most were female (n=110, 55%), single (n=100, 50%), had an undergraduate education (n=114, 57%), and were employed (n=156, 78%). Their average age was 39.18 years (SD 9.81), with the minimum age being 20 years and the maximum being 76 years.

**Table 1:** Participant characteristics obtained from the internet-based survey (N=200).

Participant characteristics			n (%)
**Gender**			
	Male	90 (45)	
	Female	110 (55)	
**Age, years**			
	20 to 29	32 (16)	
	30 to 39	78 (39)	
	40 to 49	59 (29)	
	Over 50	30 (15)	
	Not mentioned	1 (0.5)	
**Marital status**			
	Single	99 (49.5)	
	Married	93 (46.5)	
	Divorced/widowed	8 (4)	
**Education**			
	Junior high school	2 (1)	
	High school	40 (20)	
	College/vocational school	37 (18.5)	
	Undergraduate	114 (57)	
	Postgraduate	7 (3.5)	
**Individual income**			
	No income	31 (15.5)	
	<2 million yen	69 (34.5)	
	2 to 4 million yen	48 (24)	
	4 to 6 million yen	34 (17)	
	6 to 8 million yen	13 (6.5)	
	8 to 10 million yen	5 (2.5)	
**Work status**			
	Employed	155 (77.5)	
	Unemployed	45 (22.5)	
**Occupation type**			
	Managers	8 (4)	
	Specialists/technicians	31 (15.5)	
	Office work	37 (18.5)	
	Manual work	19 (9.5)	
	Service/marketing	21 (10.5)	
	Others	42 (21)	
	Unemployed	42 (21)	
**Employment contract**			
	Full-time	69 (34.5)	
	Contract worker	16 (8)	
	Temporary worker	6 (3)	
	Part-time	28 (14)	
	Self-employed	32 (16)	
	Others	6 (3)	
	Unemployed	43 (21.5)	

### Internal Consistency

Table 2 shows the mean scores of the iOSDMH for users and Cronbach α values. The mean of the total score of the iOSDMH was 51.73 (range 19-76). The Cronbach α values were slightly below the threshold (α>.7) for acceptability (α=.665), but well above the threshold for appropriateness (α=.776), feasibility (α=.832), and harm (α=.777).

**Table 2:** Average, SD, and reliability among the Japanese population for the iOSDMH and their subscales (N=200).

iOSDMH^a^ subscales (number of items; possible range)	Mean (SD)	Cronbach α
Total (19 items; 19-76)	51.73 (5.1)	.685
Acceptability (3 items; 3-12)	8.62 (2.43)	.665
Appropriateness (4 items; 4-16)	11.76 (4.21)	.776
Feasibility (6 items; 6-24)	18.84 (7.94)	.832
Harm (5 items; 5-20)	9.47 (8.64)	.777
Satisfaction (1 item; 1-4)	3.06 (0.58)	N/A^b^

^a^iOSDMH: implementation outcome scales for digital mental health.

^b^Not available.

### Factor Structure of iOSDMH

The EFA results are shown in Table 3. EFA conducted according to the Kaiser–Guttman criterion yielded 3 factors. The first factors were acceptability and appropriateness. The second was feasibility, and the third was harm. All items showed factor loadings above 0.4, so we kept them intact.

**Table 3:** Exploratory factor analysis without assuming the number of factors by using least-squares method with Promax rotation^a^.

Item number	Short description of the item	Concept	Factor loading score		
			1	2	3
6	Suitable for my social conditions	Appropriateness	*0.813*	–0.192	–0.063
5	Applicable to my health status	Appropriateness	*0.757*	–0.076	0.089
7	Fits my living condition	Appropriateness	*0.696*	–0.090	–0.130
3	Acceptable for me	Acceptability	*0.695*	0.174	–0.011
2	Improves my social image	Acceptability	*0.532*	–0.017	–0.037
1	Advantages outweigh the disadvantages for keeping my mental health	Acceptability	*0.481*	0.019	0.067
4	Appropriate (from your perspective, it is the right thing to do)	Appropriateness	*0.463*	0.325	0.074
10	Total length is implementable	Feasibility	–0.135	*0.922*	0.11
11	Length of one content is implementable	Feasibility	–0.113	*0.839*	–0.039
12	Frequency is implementable	Feasibility	0.073	*0.575*	–0.039
13	Easy to understand	Feasibility	0.163	*0.559*	–0.009
9^b^	Physical effort	Feasibility	–0.062	*0.518*	–0.24
8	Easy to use	Feasibility	0.372	*0.492*	0.124
18	Time-consuming	Harm	–0.075	–0.086	*0.723*
16	Mental symptoms	Harm	–0.009	–0.154	*0.657*
17	Induced dangerous experience regarding safety	Harm	0.051	0.227	*0.610*
15	Physical symptoms	Harm	0.086	–0.235	*0.592*
19	Excessive pressure on learning regularly	Harm	–0.077	–0.019	*0.562*

^a^Italicized values are significant.

^b^Used a reversed score.

## Discussion

### Principal Findings

This study developed implementation outcome scales for digital mental health based on existing literature and reviews by experts on web-based psychotherapy and implementation science. Our measurements included 3 key constructs of the implementation outcomes (acceptability, appropriateness, and feasibility) from previous studies and additional constructs on harm and satisfaction considered necessary in the implementation process. Implementation researchers and mental health experts agreed that each instrument of the implementation measures reflected the correct concepts.

This study created implementation outcomes for people involved in the implementation process: users, providers, and managers or policy makers. According to the World Health Organization’s implementation research guide, knowledge exchange or collaborative problem-solving should occur among stakeholders such as providers, managers or policy makers, and researchers [8]. A past study indicated that policy makers and primary stakeholders had decision frameworks that would produce different implementation outcomes [30]. Previous implementation outcome research targeted 1 or 2 groups of users, providers, and managers or policy makers. However, to our knowledge, few studies have resulted in outcome scales for different levels of stakeholders [19]. We believed that outcome measures should be adjusted for stakeholders, as decision frameworks may differ among them. For example, users of the program judge its appropriateness by considering whether it is suitable for their situations. Nevertheless, providers may find the program suitable for the circumstances of their users and for themselves. Managers or policy makers will care if the program is suitable for themselves and for users and providers. Another example is that although the length or frequency of the program may be important for feasibility among users, the cost or institutional resources may be important in assessing feasibility among managers or policy makers.

Psychometric assessment of the implementation outcomes showed good internal consistency for appropriateness, feasibility, and harm. Internal consistency for acceptability was lower than that for other constructs (α=.665), possibly because the construct for acceptability consisted of only 3 items. The EFA model suggested a 3-factor solution. The first factors were acceptability and appropriateness. Correlations between these 2 concepts were high. Our finding was consistent with previous studies in that acceptability and appropriateness were conceptually close [16,18]. For instance, it has been reported that perceived acceptability of treatment is shaped by factors such as appropriateness, suitability, convenience, and effectiveness [31,32]. However, other scholars agree that acceptability should be distinguished from appropriateness. Proctor et al stated that an individual (ie, end user) may think that an intervention that seems appropriate may not always be acceptable and vice versa [16]. Similarly, previous research on alcohol screening in emergency departments revealed that nurses and physicians found alcohol screening to be acceptable but not appropriate because the process was time-consuming, the patients might object to it, and the nurses had not received sufficient training [33,34]. Therefore, it is essential to distinguish acceptability from appropriateness in such a situation because it helps focus on the appropriate concept during implementation. Therefore, we decided to maintain the 4-factor questionnaire comprising acceptability, appropriateness, feasibility, and harm.

The strength of this study was that we selected the concepts that seemed relevant to implementation research based on literature, modified them for electronic mental health settings, and improved the contents based on discussions with expert panels. Moreover, this study developed each questionnaire for users, providers, and managers or policy makers, all having an essential role in the implementation [8]. Evaluating the implementation outcomes of different stakeholders will clarify different perceptions of the intervention program, possibly leading to active knowledge exchange among users, providers, and consumers. Although our outcome measures need further evaluation, our study contributes to implementation research in digital mental health.

We acknowledge the following limitations of our study. First, it was vulnerable to selection bias. As we recruited participants via the internet for the psychometric validation study, they might not be representative of the general population in Japan. It is possible that the participants were more familiar with web-based programs, and they may have had a better understanding of digital mental health programs. In addition, this study conducted psychometric assessment for the outcome scales for users only because the intervention setting in which interested individuals enrolled themselves in the program did not involve any providers or managers. In a study setting involving providers and managers or policy makers, the iOSDMH for providers and managers or policy makers will be needed to evaluate implementation outcomes. We plan to evaluate the iOSDMH for providers and managers or policy makers in our future intervention study (UMIN-CTR: ID UMIN 000036864). Another limitation is that criterion-related validity was not evaluated in the current psychometric assessment. The development process of the items may not be regarded as a theoretical approach. Future studies should evaluate criterion-related validity using other measures related to implementation concepts, such as the system usability scale or participation status of web-based programs. This study validated the Japanese version of the iOSDMH for users. Additional studies are needed for validating the English version. In future studies, we plan to apply these outcome measures in several web-based intervention trials to assess whether these implementation outcomes will predict the completion rate and participant attitude using digital access log information [35]. Although we tried to include multiple researchers in the digital mental health and implementation science domains from different countries, the iOSDMH scales would become more robust with a larger and more diverse review team. Finally, the setting in which we conducted the survey was an occupational setting (ie, for workers). Future studies should evaluate the scales in other settings (eg, clinical, school).

### Conclusions

We developed implementation outcome scales for digital mental health interventions to assess the perceived outcomes for users, providers, and managers or policy makers. Psychometric assessment of the outcome scale for users showed acceptable reliability and validity. Future studies should apply the newly developed measures to assess the implementation status of the digital mental health program among different stakeholders and enhance collaborative problem-solving.

## Appendix

Multimedia Appendix 1 Implementation Outcome Scales for Digital Mental Health (iOSDMH).

## Abbreviations

EFA: exploratory factor analysis
iOSDMH: implementation outcome scales for digital mental health
