# Supplementary material for: Implementation Outcome Scales for Digital Mental Health (iOSDMH): Scale Development and Cross-sectional Study
Source: JMIR Form Res. 2021 Nov 23;5(11):e24332. doi: 10.2196/24332 (PMC8663479; doi:10.2196/24332)
Supplement: Multimedia Appendix 1 [file formative_v5i11e24332_app1.docx]

**Supplementary file 1.**

**Implementation Outcome Scales for Digital Mental Health (iOSDMH) for USERs（Consumers）**

YOUR OPINION ABOUT THE PROGRAM

Please read the following statements and select ONE option that most describes your opinion about the program.

このプログラムについてのあなたの考えを教えてください。

選択肢１～４のうちあなたの考えに最も合うものを選んでください。

| 1 | 2 | 3 | 4 |
| --- | --- | --- | --- |
| Disagree | Relatively disagree | Relatively agree | Agree |
| まったくそう思わない | どちらかといえばそう思わない | どちらかといえばそう思う | そう思う |

**Part I: Evaluations of using digital mental health program**

|  | Item |
| --- | --- |
| **Acceptability 許容性** | |
| 1 | The advantages of my using this program outweigh the disadvantages for keeping my healthy mental health.  私はこのプログラムを使わないよりも使ったほうが、自分の心の健康を保つうえで役に立つ。 |
| 2 | Using this program improves my social image.  このプログラムを使用することで自分の社会的イメージが良くなる気がする。 |
| 3 | This program is acceptable for me.  このプログラムは、私にとって受け入れやすい。 |
| **Appropriateness 適切性** | |
| 4 | The content of the program is appropriate (from your perspective, it is the right thing to do).  このプログラム内容は適切である。（私からみて、この内容は正当だと思う） |
| 5 | This program is applicable with my health status (e.g. pregnancy, physical and mental condition, etc).  このプログラムは私の健康状態（例：妊娠、身体や心の状態など）に合っている。 |
| 6 | This program is suitable for my social conditions (e.g., work, housekeeping, commute, etc).  このプログラムは私の社会的な状況（例：仕事、家事、通勤など）に合っている。 |
| 7 | This program fits with my living condition (e.g., place of residence).  このプログラムは私の生活状況（例：居住地など）に合っている。 |
| **Feasibility 実施可能性** | |
| 8 | I believe this program is easy to use.  このプログラムは使いやすい。 |
| 9 | My using this program requires me physical effort (e.g., tired eyes, shoulder stiffness).  このプログラムの使用には身体的な負担がある。（＊reversed item 逆転項目） |
| 10 | The total length of the program is implementable.  プログラム全体の長さは実施可能である。 |
| 11 | The length of 1 content is implementable.  1回分の内容の長さは実施可能である。 |
| 12 | The frequency of providing program is implementable.  プログラムが提供される頻度は実施可能である。 |
| 13 | The program is easy to understand.  このプログラムはわかりやすい。 |
| **Overall Satisfaction　全体の満足度** | |
| 14 | Overall, I am satisfied with the program.  全体として、私はこのプログラムに満足している。 |

**Part II: Adverse events of using digital mental health program**

|  | Item |
| --- | --- |
| **Harms 有害事象** | |
| 1 | Using this program causes physical symptoms (e.g.,. Tired eyes, headache, stiffness shoulders)  このプログラムを使用することで身体的な症状が起きる。（例：目が疲れる、頭痛、肩こりなど） |
| 2 | Using this program causes mental symptom (e.g., depression, insomnia).  このプログラムを使用することで精神的な症状が起きる。（例：落ち込む、不眠） |
| 3 | Using this program sometimes brings us a smart phone induced dangerous experience regarding safety (e.g., collide with people while walking and looking at the smart phone).  このプログラムをスマートフォンで使用することで、危険な経験をする。（例：歩きながら使っていて他の人とぶつかる） |
| 4 | I have a concern that the use of this program consumes my time for other activities（e.g., time for leisure, family affairs, sleep, education）  私はこのプログラムの使用によって自分の時間が削られているのではないかと気になる。（例：余暇、家族、睡眠、自己学習の時間） |
| 5 | Using this program makes me face the excessive pressure on learning this program regularly.  このプログラムを定期的に使わなければというプレッシャーを感じて負担になる。 |

**Implementation Outcome Scales for Digital Mental Health (iOSDMH) for PROVIDERs***

*PROVIDERs refer to people who have direct contact with users (e.g., medical: nurse, workplace: person in charge)

YOUR OPINION ABOUT THE PROGRAM

Please read the following statements and select ONE option that most describes your opinion about the program.

このプログラムについてのあなたの考えを教えてください。

選択肢１～４のうちあなたの考えに最も合うものを選んでください。

プログラムが使用されたセッティング: [臨床 / 行政 / 地域 / 職場 / 学校 / その他( )]

このプログラムにおけるあなたの役割：【　　　　　】

このプログラムの種類：　１．予防目的 ２．治療目的

The setting the program has been used for: [clinical / public administration / community / workplace / school / others ( )]

Your role of the program: [ ]

Type of the program: 1. Preventive 2. Therapeutic

| 1 | 2 | 3 | 4 | 5 |
| --- | --- | --- | --- | --- |
| Disagree | Relatively disagree | Relatively agree | Agree | Don’t know |
| まったくそう思わない | どちらかといえば  そう思わない | どちらかといえば  そう思う | そう思う | わからない |

**Part I: Evaluations of using digital mental health program**

|  | Item |
| --- | --- |
| **Acceptability 許容性** | |
| 1 | I find this program acceptable for keeping healthy mental health of users.  ユーザーは、このプログラムは心の健康を保つのに適切だと思うだろう。 |
| 2 | I would like to serve this program in my workplace.  私は、自分の職場でこのプログラムを提供したい。 |
| 3 | Providing the program improves my evaluation in the organization.  このプログラムを提供することで、私の職場での評価が高まる。 |
| **Appropriateness 適切性** | |
| 4 | The content of the program is appropriate for a variety of users (from your perspective, it is the right thing to do).  このプログラム内容は自分がサービスの提供をするさまざまなユーザーにとって適している。（私からみて、この内容は正当だと思う） |
| 5 | This program is compatible with my current situation (e.g. type of institution, location of your work, your resources, etc).  このプログラムは私の現在の状況（例：所属機関、地域、忙しさ、資源、就労状況など）に合っている。 |
| 6 | This program is suitable for situations of users (health status, and social condition, living condition, etc).  このプログラムはユーザーの状況（例：健康状態、社会的な状況、生活状況）に合っている。 |
| **Feasibility 実施可能性** | |
| 7 | I believe this program is easy to use for users.  このプログラムはユーザーにとって使いやすい。 |
| 8 | It is easy to provide this program to users.  私にとって、この手法をユーザーに提供するのは簡単だ。 |
| 9 | Providing feedback for users of this program （e.g.answering questions, facilitating users for more participation, etc) is implementable.  私にとって、ユーザーにこのプログラムのフィードバック（例：質問に対応する、積極的な参加を促すなど）を行うことは、実施可能である。 |
| **Overall Satisfaction　全体の満足度** | |
| 10 | Overall, I am satisfied with the program.  全体として、私はこのプログラムの提供として、このプログラムに満足している。 |

**Part II: Adverse events of using digital mental health program**

|  | Item |
| --- | --- |
|  | **Harms 有害事象** |
| 11 | This program does not result in negative side effects for users.  このプログラムは、ユーザーに対して悪影響を及ぼすことはないだろう。 |

**Implementation Outcome Scales for Digital Mental Health (iOSDMH) for Managers or Policy makers＊**

* Managers or Policy makers refer to people who have authority to make a decision on implementation of this program (e.g., responsible person)

YOUR OPINION ABOUT THE PROGRAM

Please read the following statements and select ONE option that most describes your opinion about the program.

このプログラムについてのあなたの考えを教えてください。

選択肢１～４のうちあなたの考えに最も合うものを選んでください。

プログラムが使用されたセッティング: [臨床 / 行政 / 地域 / 職場 / 学校 / その他( )]

このプログラムにおけるあなたの役割（例．意思決定者）：【　　　　　】

このプログラムの種類：　１．予防目的 ２．治療目的

The setting the program has been used for: [clinical / public administration / community / workplace / school / others ( )]

Your role of the program: [ ]

Type of the program: 1. Preventive 2. Therapeutic

| 1 | 2 | 3 | 4 | 5 |
| --- | --- | --- | --- | --- |
| Disagree | Relatively disagree | Relatively agree | Agree | Don’t know |
| まったくそう思わない | どちらかといえば  そう思わない | どちらかといえば  そう思う | そう思う | わからない |

**Part I: Evaluations of using digital mental health program**

|  | Item |
| --- | --- |
| **Acceptability 許容性** | |
| 1 | This program is acceptable for our institution.  このプログラムは、この組織にとって受け入れやすい。 |
| 2 | Using this program gives our institution a positive social image.  このプログラムを使うことで組織の社会的なイメージが良くなる。 |
| 3 | This program is acceptable for providers.  このプログラムは、プログラムを提供するスタッフにとって受け入れやすい。 |
| 4 | This program is acceptable for users.  このプログラムは、ユーザーにとって受け入れやすい。 |
| **Appropriateness 適切性** | |
| 5 | This program fits into our brand image.  このプログラムは私たちの組織のブランドイメージに合っている。 |
| 6 | This program fits with issues our institution should work for.  このプログラムは私たちの組織が取り組むべき課題と合っている。 |
| 7 | This program is compatible with the current situation of our institution. (e.g, ex. the number of staff, busyness of staff）  このプログラムは私たちの組織の現在の状況に合っている。（例：スタッフ数やスタッフの忙しさ） |
| 8 | This program is appropriate for users (consumers).  このプログラムはユーザー（実際に利用する人）にとって有効だと思う。 |
| **Feasibility 実施可能性** | |
| 9 | This program is implementable in regard of cost in our institution.  コストの面で、このプログラムは私たちの組織で実施可能である。 |
| 10 | This program is implementable in regard and resources in our institution (e.g., staff, network environment).  資源の面で（例：スタッフ、通信環境）、このプログラムは私たちの組織で実施可能である。 |
| 11 | Using this program requires providers a lot of effort.  このプログラムを使うことでプログラムを提供するスタッフにかなり負担がある。 |
| 12 | Using this program requires users a lot of effort.  このプログラムを使うことでユーザーにかなり負担がある。 |
| **Overall Satisfaction　全体の満足度** | |
| 13 | Overall, I am satisfied with the program.  全体として、私はこのプログラムに満足している。 |

**Part II: Adverse events of using digital mental health program**

|  | Item |
| --- | --- |
| **Harms 有害事象** | |
| 14 | This program does not result in negative side effects (e.g., physical or psychological symptoms).  このプログラムは、ユーザーに対して悪影響を及ぼすことはないだろう（例：身体的、精神的な負担）。 |
